# Phosphoglycerate dehydrogenase is dispensable for breast tumor maintenance and growth

**DOI:** 10.18632/oncotarget.1540

**Published:** 2013-11-26

**Authors:** Jinyun Chen, Franklin Chung, Guizhi Yang, Minying Pu, Hui Gao, Wei Jiang, Hong Yin, Vladimir Capka, Shailaja Kasibhatla, Bryan Laffitte, Savina Jaeger, Raymond Pagliarini, Yaoyu Chen, Wenlai Zhou

**Affiliations:** ^1^ Oncology, Novartis Institutes for Biomedical Research, Cambridge, Massachusetts, United States; ^2^ Analytic Science, Novartis Institutes for Biomedical Research, Cambridge, Massachusetts, United States; ^3^ The Genomics Institute of the Novartis Research Foundation, San Diego, California, United States

**Keywords:** PHGDH, breast cancer cells, *in vivo*

## Abstract

Cancer cells rely on aerobic glycolysis to maintain cell growth and proliferation via the Warburg effect. Phosphoglycerate dehydrogenase (PHDGH) catalyzes the first step of the serine biosynthetic pathway downstream of glycolysis, which is a metabolic gatekeeper both for macromolecular biosynthesis and serine-dependent DNA synthesis. Here, we report that PHDGH is overexpressed in many ER-negative human breast cancer cell lines. PHGDH knockdown in these cells leads to a reduction of serine synthesis and impairment of cancer cell proliferation. However, PHGDH knockdown does not affect tumor maintenance and growth in established breast cancer xenograft models, suggesting that PHGDH-dependent cancer cell growth may be context-dependent. Our findings suggest that other mechanisms or pathways may bypass exclusive dependence on PHGDH in established human breast cancer xenografts, indicating that PHGDH is dispensable for the growth and maintenance of tumors *in vivo*.

## INTRODUCTION

**Fig 1:**
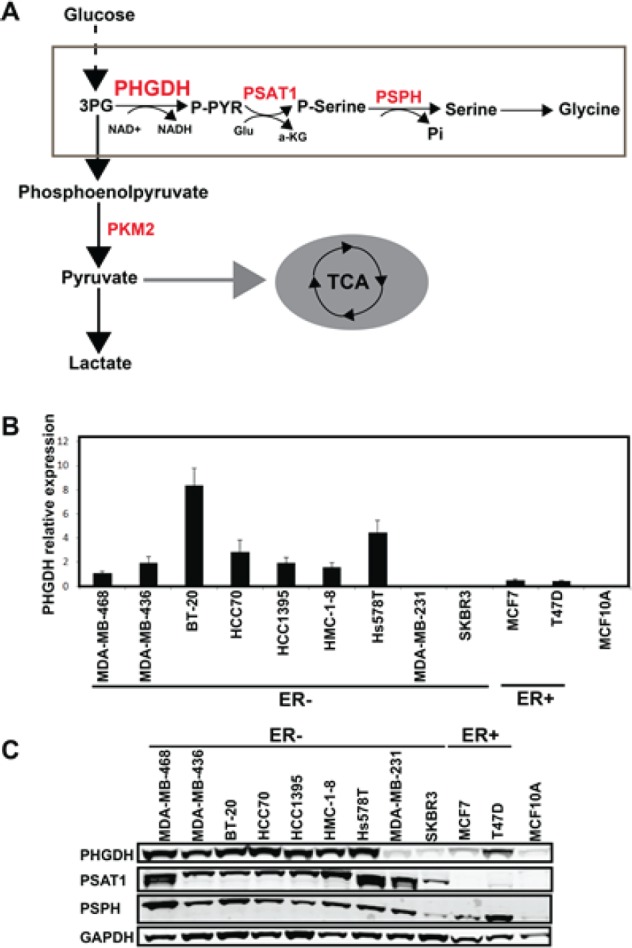
*PHGDH* shows a differential expression pattern among human breast cancer cell lines

The Warburg effect is defined as aerobic glycolysis and used by cancer cells to maintain cell growth and proliferation[1]. Increased rates of glucose uptake, with a decrease in oxidative phosphorylation even in the presence of oxygen, are often observed in many types of cancer cells [1, 2]. *PHGDH* encodes 3-phosphoglycerate dehydrogenase, which is the first enzyme branching from glycolysis into the serine synthetic pathway (Fig.1A) [3]. It requires nicotinamide adenine dinucleotide (NAD) as a cofactor to oxidize the glycolytic intermediate 3-phosphoglycerate into phospho-hydroxypyruvate [4, 5]. The serine production pathway also includes two subsequent enzymes: phosphoserine aminotransferase 1 (PSAT1) and phosphate ester hydrolysis (PSPH) (Fig. 1A) [3]. Serine is needed for synthesis of proteins and other biomolecules in cell proliferation and its biosynthetic activity was shown to be elevated in tumor lysates [6, 7].

A. The schematic of diversion of glucose metabolism into serine and glycine metabolism through *PHGDH*. B. The mRNA levels of *PHGDH* were measured among different human breast cancer cell lines. Total RNA was isolated and real-time PCR analysis was performed. Taqman probe of target gene and B2M transcripts were coamplified. The mRNA expressions of target genes were normalized to B2M levels. All experiments were performed in triplicate. C. Western blotting analysis of human breast cancer cells. Cancer cell samples were collected from different human breast cancer cell lines. The protein lysates were prepared and western blotting analysis of *PHGDH*, PSAT1, PSPH and GAPDH were performed.

Recently, by taking advantage of *in vivo* RNA interference (RNAi)-based loss-of-function screening in a human MCF10DCIS.COM cancer cells, *PHGDH* was identified as one of several genes necessary for the growth of tumor cells [8]. In primary breast tumors, *PHGDH* localizes to a genomic region of recurrent copy number gain and its protein levels are elevated in 70% of estrogen receptor (ER)-negative breast cancers [8]. Suppression of PHGDH in PHGDH high-expression cancer cell lines causes a strong decrease in cell proliferation, as well as a reduction in serine synthesis [8, 9]. Besides breast cancer, *PHGDH* is also amplified in human melanoma and PHGDH knockdown impairs proliferation of those melanoma cells [9, 10]. In addition, PHGDH and PSAT expression levels are elevated in human intestinal tumors with deficiency of Protein kinase C(PKC)ζ, which promotes the plasticity necessary for cancer cells to use glutamine through the serine biosynthesis pathway in the absence of glucose[11]. These findings suggest that the PHGDH regulating diversion of metabolism pathways may be important during tumor development. Therefore, targeting the serine synthesis pathway may be therapeutically valuable in breast cancers with elevated PHGDH expression or amplifications [8].

While recent studies showed the requirement of PHGDH in human breast tumor initiation, the role of PHGDH in established breast tumors is not fully understood. Here, we show that PHGDH is overexpressed in ER-negative human breast cancer cells and *PHGDH* knockdown impairs the proliferation of those cells *in vitro*. However, PHGDH is dispensable for breast tumor maintenance and growth *in vivo*, suggesting that requirement of PHGDH is context-dependent.

## RESULTS

### PHGDH shows a differential expression pattern among human breast cancer cell lines

To gain insight into whether PHGDH, the key enzyme in the glucose metabolism pathway (Fig. 1A), is necessary for breast cancer cell proliferation, we first examined the expression pattern of PHGDH in eleven human breast cancer cell lines with varying ER status. Most, but not all, ER^−^ breast cancer cells show high-level expression of PHGDH. PHGDH is also modestly expressed in ER^+^ T47D cells but is barely detected in ER^+^ MCF7, two ER^−^ lines (MDA-MB-231 and SKBR3), and the non-transformed breast epithelial cell line MCF10A (Fig.1 A and B). Interestingly, PSAT1 and PSPH, the other two enzymes involved in serine biosynthesis, are also highly expressed in most ER^−^ breast cancer cells (Fig.1C). As PHGDH amplification is associated with significant protein overexpression in primary human breast cancer and melanoma [9], we next assessed *PHGDH* copy number in these breast cancer cell lines. We found that apart from BT20, with 5 copies of *PHGDH*, other breast cancer cell lines did not show high-level copy number gain of *PHGDH* (Supplementary Fig.1). These findings suggest that enzymes of the PHGDH are overexpressed in most ER^−^ breast cancers, but there is no definitive correlation with ER^−^ status, and this overexpression is not commonly driven via genetic means such as DNA copy number alterations.

### PHGDH knockdown impairs the proliferation of breast cancer cells with PHGDH overexpression *in vitro*

**Fig 2:**
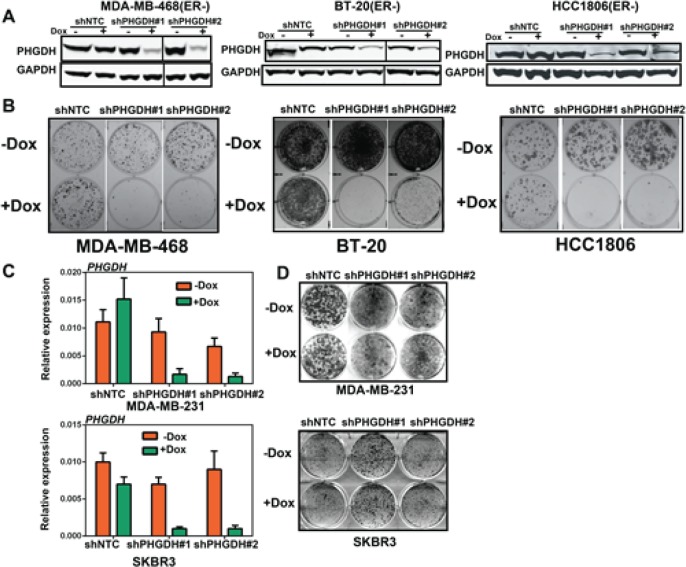
*PHGDH* knockdown impairs the proliferation of breast cancer cells with *PHGDH* overexpression *in vitro*

Our findings, as well as those from other groups [8], suggest that PHGDH is overexpressed in a subset of ER^−^ breast cancer cell lines. Next, we tested whether breast cancer cells with PHGDH overexpression were sensitive to *PHGDH* knockdown. Two Doxycycline inducible shRNA constructs targeting distinct sequences in *PHGDH* were stably introduced into three PHGDH pathway low-expression breast cancer cell lines: MDA-MB-231, SKBR3 and T47-D; five PHGDH pathway high expression breast cancer cell lines: MDA-MB-468, BT-20, HCC1395, HCC1806 and HCC70; and one non-transformed breast cell line: MCF10A (Table.1). When shRNA expression was induced by Doxycycline, robust *PHGDH* knockdown was achieved in breast cancer cell lines at both the mRNA and protein levels (Table. 1, Fig. 2A and 2C).

**Table 1:** PHGDH knockdown impairs PHGDH high expression breast cancer cells proliferation *in vitro* The effect of *PHGDH* knockdown was observed among 8 breast cancer cell lines and 1 normal breast cell line.

Genotype	Cell Lines	PHGDH KD at Day 5		GI* by CTG at Day 7		GI by CFA at Day 14	
		shPHGDH#1	shPHGDH#2	shPHGDH#1	shPHGDH#2	shPHGDH#1	shPHGDH#2
**PHGDH high** TNBC, Basal-like	MDA-MB-468	97%	94%	57%	0%	+++	+++
	BT20	96%	93%	85%	3%	++++	+++
	HCC1395	90%	84%	51%	0%	+++	+++
	HCC1806	97%	92%	51%	29%	++++	++++
	HCC70	92%	93%	No effect		−	+
**PHGDH Low.** ER-	MDA-MB-231	85%	89%	No effect		No effect	
	SKBR3	89%	91%	No effect		No effect	
**PHGDH Low.** ER+	T47-D	80%	80%	No effect		No effect	
**Non-transformed**	MCF10A	82%	73%	No effect		No effect	++

* **GI: growth inhibition**

++++ **: 100% GI,**

+++ **: 75% GI,**

++ **: 50% GI,**

+ **: 25% GI,**

− **: 0% GI**

A. Western blotting analysis of *PHGDH* knockdown in MDA-MB-468, BT-20 and HCC1806 cells. *shNTC* or *shPHGDH* transduced stable cell lines were treated with Doxycycline for 3 days and cell pellets were collected and western blotting was performed. B. Cell colony formation assay of *PHGDH* knockdown in MDA-MB-468, BT-20 and HCC1806 cells. *shNTC* or *shPHGDH* transduced cancer cells were seeded at low density in individual wells of a standard 6-well plate and grown in normal serum medium with or without Doxycycline for 14 days. Cancer cells colonies were visualized by crystal violet staining. C. Real-time PCR analysis of *PHGDH* knockdown in MDA-MB-231 and SKBR3 cells. *shNTC* or *shPHGDH* transduced stable cell lines were treated with or without Doxycycline for 3 days and cell pellets were collected. Total RNA was isolated and real-time PCR analysis was performed. Taqman probes of target genes and B2M transcripts were coamplified. The mRNA expressions of target genes were normalized to B2M levels. All experiments were performed in triplicate. D. Cell colony formation assay of *PHGDH* knockdown in MDA-MB-231 and SKBR3 cells. *shNTC* or *shPHGDH* transduced cancer cells were seeded at low density in individual wells of a standard 6-well plate and grown in normal serum medium with or without Doxycycline for 14 days. Cancer cells colonies were visualized by crystal violet staining.

We next tested whether *PHGDH* knockdown affects the proliferation of these cell lines in colony formation assays. *PHGDH* knockdown significantly inhibited the growth of MDA-MB-468, BT-20 and HCC1806 *in vitro* (Fig. 2B). Short-term cell proliferation assays also showed that *PHGDH* knockdown inhibited the growth of HCC1806 (Supplementary Fig.2). *PHGDH* knockdown also showed a significant inhibition on cell proliferation in HCC1395 but not HCC70 (Table.1). In contrast, colony formation assays showed that *PHGDH* knockdown didn't affect the growth of MDA-MB-231 and SKBR3 (Fig. 2D), as well as T47D and MCF10A (Table. 1) *in vitro*. Collectively, robust *PHGDH* knockdown did not affect non-transformed breast cells, ER^+^ breast cancer cells and ER^−^ breast cancer cells with low coordinate expression of PHGDH pathway components. Therefore, *PHGDH* knockdown selectively affects the growth of ER^−^ breast cancer cells with PHGDH high-expression.

### Sensitivity to PHGDH knockdown is tightly associated with effects on the production of glucose-derived serine and glycine

**Fig 3:**
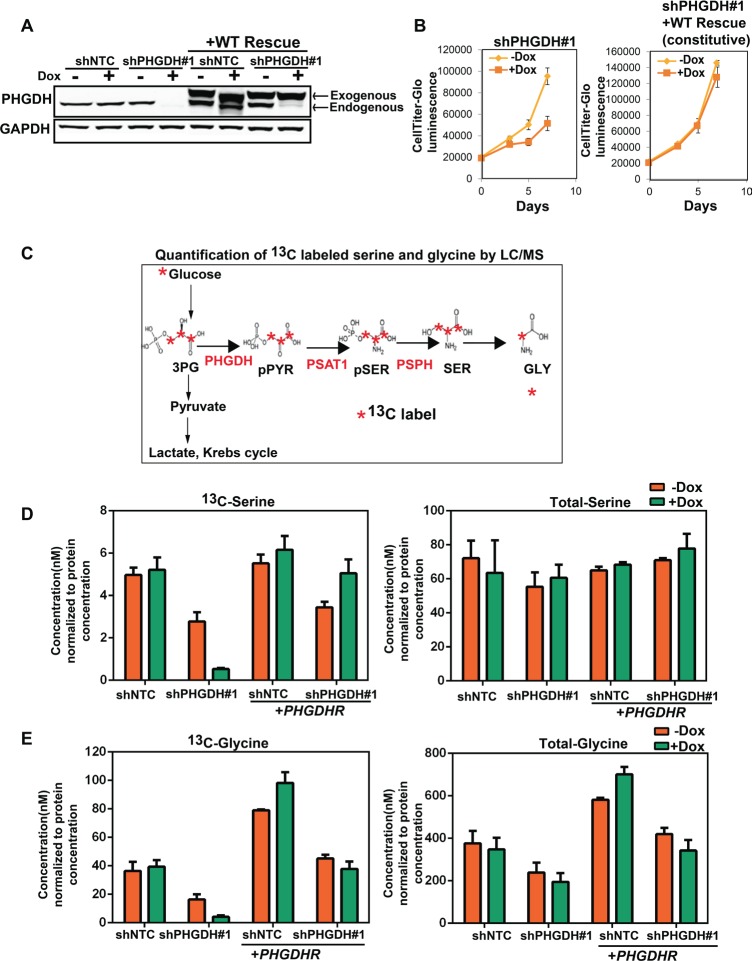
Sensitivity to *PHGDH* knockdown is tightly associated with effects on the production of glucose-derived serine and glycine

To evaluate whether the phenotype mediated by *PHGDH* shRNA knockdown was on-target, RNAi-resistant *PHGDH* (*PHGDHR*) cDNA was constitutively expressed in MDA-MB-468 cells with inducible *PHGDH* knockdown. Expression of *PHGDHR* cDNA restored the expression of PHGDH in MDA-MB-468 cells when endogenous PHGDH expression was depleted by Doxycycline treatment(Fig. 3A) and rescued the cell proliferation phenotype medicated by *PHGDH* knockdown (Fig. 3B), which indicates that the inhibitory effects of *PHGDH* knockdown on the proliferation of breast cancer cells is on-target.

A. Western blotting analysis of MDA-MB-468 cells expressing the indicated shRNA and cDNA contruct. The constitutive lenti-virus construct (*PHGDH*R cDNA) was used to transduce MDA-MB-468 cells and expression of *PHGDH*R cDNA with or without *PHGDH* knockdown were tested. *PHGDH*R cDNA overexpressed MDA-MB-468 cells with or without *PHGDH* knockdown were treated with Doxycycline for 5 days and cell pellets were collected and *PHGDH* and GAPDH were detected by western blotting. B. The proliferation of MDA-MB-468 cells with inducible expression of either *PHGDH* shRNA alone or both *PHGDH* shRNA and *PHGDH*R cDNA(constitutive or inducible expression). *PHGDH* shRNA alone or both *PHGDH* shRNA and *PHGDH*R cDNA transduced cancer cells were treated with or without Doxycycline for different days. Relative cell growth (average of at least 3 independent experiments) was measured by CellTiter-Glo. C. The schematic of labeled serine and glycine quantification by LC/MS. D. Quantification of 13C -serine and total serine in MDA-MB-468 cells expressing the indicated shRNA and inducible cDNA construct. The inducible lenti-virus construct (*PHGDH*R cDNA) was used to transduce MDA-MB-468 cells and expression of *PHGDH*R cDNA with or without *PHGDH* knockdown were tested. *PHGDH*R cDNA overexpressed MDA-MB-468 cells with or without *PHGDH* knockdown were treated with Doxycycline for 5 days, then followed by treatment of cell culture medium with or without 13C-glucose for 24 hours. Cell pellets were collected and 13C-serine and total serine were detected by LC/MS. E. Quantification of 13C-glycine and total glycine of MDA-MB-468 cells expressing the indicated shRNA and inducible cDNA construct. The inducible lenti-virus construct (*PHGDH*R cDNA) was used to transduce MDA-MB-468 cells and expression of *PHGDH*R cDNA with or without *PHGDH* knockdown were tested. *PHGDH*R cDNA overexpressed MDA-MB-468 cells with or without *PHGDH* knockdown were treated with Doxycycline for 5 days, then followed by treatment of cell culture medium with or without 13C-glucose for 24 hours. Cell pellets were collected and 13C-glycine and total glycine were detected by LC/MS.

We next sought to investigate the effects of PHGDH modulation on serine biosynthetic phenotypes. To address this, cells were cultured in the presence of ^13^C-labeled glucose in order to trace of its contribution to newly synthesized serine or glycine (Fig. 3C). *PHGDH* knockdown reduced the production of glucose-derived serine and glycine. *PHGDHR* cDNA restored the production of glucose-derived ^13^C-serine and ^13^C-glycine in these cells (Fig. 3D and Fig. 3E). These data show a good correlation of the growth effects with inhibition of serine biosynthetic pathways, suggesting that growth inhibition is mediated through these pathways.

While glucose derived serine was depleted upon PHGDH knockdown, total serine levels did not appear to be dramatically changed (Fig. 3D). To determine if PHGDH knockdown effects were due to global serine depletion, we increased the levels of serine or methyl-serine in cell culture medium, but this was insufficient to rescue PHGDH knockdown-mediated growth inhibition of BT-20 (Supplementary Fig.3), suggesting that ER- breast cancer cells depend on a specific function of PHGDH and glucose-derived serine and glycine production, or alternately that these cells rely on PHGDH to perform a function independent of serine and glycine production.

### PHGDH knockdown does not affect tumor growth in xenograft mouse models

**Fig 4:**
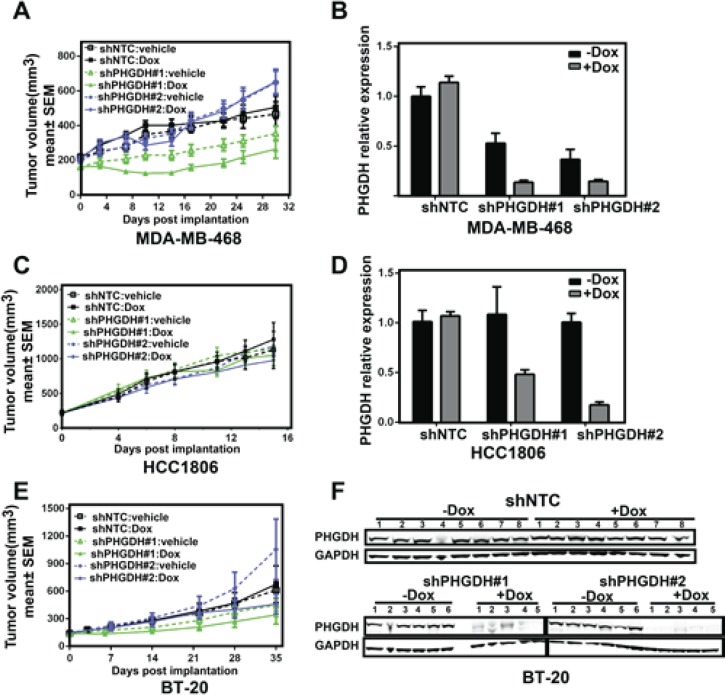
*PHGDH* knockdown does not affect tumor growth in xenograft mouse models

To further understand the role of PHGDH in tumor growth and maintenance *in vivo*, nude mice were injected subcutaneously with MDA-MB-468, HCC1806 or BT-20 cells expressing Doxycycline-inducible shRNA to *PHGDH.* In the MDA-MB-468 xenograft mouse model, one *PHGDH* shRNA showed some growth effects, but this didn't correlate with *PHGDH* knockdown, raising the concern that the growth effect observed was due to off target of this hairpin in the 468 cell line (Fig. 4A and B). The other *PHGDH* shRNA did not inhibit tumor growth significantly (Fig. 4A), although robust *PHGDH* knockdown was achieved (Fig. 4B). Similar to the MDA-MB-468 xenograft mouse model, *PHGDH* shRNA also did not inhibit tumor growth significantly in HCC1806 and BT-20 xenograft mouse models (Fig. 4C and E), although robust *PHGDH* knockdown was also achieved in either the mRNA or protein level (Fig. 4D and F). These results demonstrate that, although a dependence of breast cancer cell proliferation on PHGDH activity was found *in vitro,* PHGDH is not required for breast tumor maintenance and growth *in vivo*.

A. The effect of *PHGDH* knockdown in MDA-MB-468 xenograft mouse model. Tumor growth rate of MDA-MB-468 cells expressing inducible control shRNA or shRNA against *PHGDH* under Doxycycline were compared at different time points. B. Real-time PCR analysis of tumor samples. Tumor samples were collected at the end of studies (30 days post Doxycycline treatment). Total RNA was isolated from tumor samples and real-time PCR analysis of *PHGDH* mRNA was performed. C. The effect of *PHGDH* knockdown in HCC1806 xenograft mouse model. Tumor growth rate of HCC1806 cells expressing inducible control shRNA or shRNA against *PHGDH* under Doxycycline were compared at different time points. D. Real-time PCR analysis of tumor samples from HCC1806 xenograft mouse model. Tumor samples were collected after 7 days post Doxycycline treatment. Total RNA was isolated from tumor sample and real-time PCR analysis of *PHGDH* was performed. E. The effect of *PHGDH* knockdown in BT-20 xenograft mouse model. Tumor growth rate of BT-20 cells expressing inducible control shRNA or shRNA against *PHGDH* under Doxycycline were compared at different time points. F. Western blotting analysis of tumor samples. Tumor samples were collected at the end of studies (35 days post Doxycycline treatment) and protein lysates were prepared and western blotting analysis of *PHGDH* and GAPDH were performed

## DISCUSSION

We observe that *PHGDH* knockdown decreases the production of serine and glycine and impairs glucose metabolism in ER^−^ breast cancer cell lines with overexpression of PHGDH and inhibits the proliferation of these breast cancer cells *in vitro*. However, *PHGDH* knockdown does not affect the growth of three of these models when assessed as fully established breast cancer xenografts in mice. Previous work has shown that PHGDH is required for breast tumor initiation and growth in MCF10DCIS.com and MDA-MB-468 *in vivo* models [8], which is ostensibly in contrast with our results. This discrepancy may be due to differences in tumor sizes used when assessing PHGDH knockdown. Prior work employed PHGDH knockdown in relatively small tumors compared with those used in our study. It is conceivable that smaller tumors may replicate more of a “tumor initiation” stage relative to larger tumors, which may be more relevant to studying the maintenance of fully established tumors. *PHGDH* inducible knockdown was started at relative smaller tumor size in both MCF10DCIS.com and MDA-MB-468 *in vivo* models, which may be more relevant to the tumor initiation status [8]. The different effects of *PHGDH* knockdown on tumor initiation and maintenance suggest that the requirement of PHGDH in cancer is context-dependent or tumor stage dependent.

In addition to *PHGDH*, other glucose metabolism genes have also been shown to be more important for tumor initiation than tumor maintenance. The glycolytic pyruvate kinase isoenzyme M2 (PKM2) determines whether glucose is converted to lactate for regeneration of energy (active form, Warburg effect) or used for the synthesis of cell building blocks in tumor cells[12]. PKM2 shows a predominant expression in tumors and overexpression of PKM2 mutant reduced glycolysis and led to decreased tumor initiation and growth [13-16]. However, like PHGDH, PKM2 is shown to be dispensable for tumor maintenance and growth in xenograft mouse models [17, 18]. These observation raises a very interesting question: why PHGDH- and PKM2- dependency are only observed in specific setting, for example: colony formation and tumor initiation? Why might tumors no longer require PHGDH or PKM2 when they become bigger or relative later stage? One possible reason is that the reduction of metabolic products of PHGDH *in vivo* might be restored by other metabolism pathways when tumor sizes increase or tumors enter into later stages. Recent studies indicate that metabolic pathways undergo dramatic changes during tumor development. For example, a metabolic shift, including downregulation of genes involved in the TCA cycle, upregulation of the pentose phosphate pathway and the glutamine transporter genes and increased acetyl-CoA carboxylase protein, correlates with clear cell renal cell carcinoma tumor stages [19].

In light of recent findings, serine was found to be able to bind to and activate the glucose metabolism enzymes such as PKM2 [20]. Serine deprivation reduces PKM2 activity and shifts cells to a fuel-efficient mode [20]. Other metabolism pathways activated by serine might override the function of PKM2. Cancer dependence of PKM2 is found in serine depleted condition, which suggests that PKM2 activators may only work in patients with nutrition restriction [21]. It would be interesting to determine whether PKM2 activation could combine with PHGDH inhibition to have more dramatic effects on the growth of established ER- breast cancer models.

Our results suggest that global serine/glycine level changes are not the reason for PHGDH dependence in PHGDH-dependent cells, which is consistent with a recent study from Possemato et.al [8]. Their study suggested that serine production from the glycolytic intermediate mediated by PHGDH/PSAT1/PSPH pathway is unlike to be the reason of the cancer dependency of PHGDH *in vitro* [8]. Instead, the production of α-KG by utilization of glutamine coupled to the conversion of phosphorus-hydroxypyruvate to phosphoserine by PSAT1 was thought to be critical for tumor growth [8]. Loss of PHGDH cancer dependence may be due to α-KG level restored by other pathways, such as alanine aminotransferase [8]. A better understanding of the mechanisms governing PHGDH dependence in *the in vivo* context could lead to new targets that, when inhibited, unmask functional dependence on PHGDH in established tumors.

Recent study suggests that PHGDH expression level is high in astrocytic tumors and inhibition of PHGDH in glioma cells significantly decreased cell proliferation, invasion and tumorigenicity [22]. PHGDH may be used as a potential prognostic marker for glioma patient cumulative survival [22]. The functional role of PHGDH in this setting is independent of its role in serine biosynthesis, instead as a modulator of FOXM1 protein stability [22]. However, it is not clear how PHGDH may affect the FOXM1 N-terminal induced degradation process. Interestingly, PKM2 recently was found playing a non-metabolic role in tumorigenicity by regulating beta-catenin transactivation upon EGFR activation in cancer cells [23]. These data suggest that metabolic enzymes, like PHDGH and PKM2, might have a novel function independent of their role in metabolism.

Given the complexities of understanding metabolic function in cancer cell lines and xenografts, knock-in mouse models may be an alternative choice to understand the role of metabolism gene in tumor initiation and maintenance, as the genetic background of those tumor models are less complicated. For instance, Hexokinase2 (HK2), an enzyme catalyzing the first committed step of glucose metabolism, has recently been shown to be required for tumor initiation and maintenance by using mouse models of KRas-driven lung cancer and ErbB2-driven breast cancer with *Hk2* conditional knockout [24]. To further understand how PHGDH and PKM2 regulate tumor development, PHGDH or PKM2 inducible knockout/knock-in mouse models could be further developed and crossed with mouse tumor models in order to further understand the role of those genes in tumor initiation and maintenance.

## METHODS AND MATERIALS

### Cell culture

MDA-MB-468, MDA-MB-436, BT-20, HCC70, HCC1395, HMC-1-8, Hs578T, MDA-MB-231, SKBR3, MCF7, T47D and HCC1806 cells were obtained from American Type culture Collection. All cell lines were maintained in Dulbecco's Modification of Eagle's Medium, McCoy's 5a medium or advanced RPMI medium 1640 (Invitrogen) with 10% FBS (Invitrogen). Infected cell lines were maintained under 1 μg/mL of puromycin (MP Biomedicals) for selection. MCF-10A cells were cultured as described previously[25].

### Short hairpin RNA and RNAi-resistant constructs

Control short hairpin RNA (shRNA), GGATAATGGTGATTGAGATGG, PHGDH shPHGDH#1, CTTCGATGAAGGACGGCAAAT, and PHGDH shPHGDH#2, CAGCAATAACCGTCTAATAAA, were cloned into the inducible pLKO-Tet-On puromycin vector as previously described[26, 27]. RNAi-resistant PHGDH cDNA was ordered from DNA2.0 and cloned into pLKO-Trex vector.

### Lentivirus and infection

Lentiviral supernatants were generated according to our previously established protocol [27]. A total of 100 μL of lentivirus was used to infect 300,000 cancer cells in a six-well plate, in 8 μg/mL polybrene (Chemicon). Medium was replaced and after 24 h, cells were selected by puromycin (MP Biomedicals) and expanded. Induction of shRNA was obtained by addition of 100ng/mL Doxycycline (Clontech) to the medium.

### RNA extraction and quantitative Reverse Transcription-PCR

Total RNA was isolated using the RNeasyMini kit (Qiagen). ABI taqman gene expression and VICMGB primers/probe sets (Applied Biosystems) were used in each reaction to coamplify the B2M transcripts. All experiments were performed in triplicate and normalize to B2M levels as indicated.

### Western blotting

Western blotting was performed as follows: total tumor lysates were separated by SDS/PAGE and electrotransferredto nitrocellulose membrane (Invitrogen). Membraneswere blocked in PBS and 0.1% (vol/vol) Tween-20 (PBS-T) and 5% (wt/vol) nonfat dry milk (Bio-Rad) for 1 h on a shaker at room temperature. Primary antibodies were added to the blocking solution at 1:1,000 (PHGDH; Abcam 102789), 1:1,000 (PSAT1; Thermo PA5-22124), 1:1,000(PSPH; Thermo PA5-22003) and 1:10,000 (GAPDH; Cell Signaling Technology, 2118S) dilutions and incubated overnight and a rocker at 4 °C. Immunoblottings were washed three times, 5 min each with PBS-T, and secondary antibody was added at 1:10,000 dilution into PBS-T milk for 1 h on a shaker at room temperature. After several washes, enhanced chemiluminescence (ECL) reactions were performed according to manufacturer's recommendations (SuperSignal West Dura Extended Duration Substrate; Thermo Scientific).

### Cell viability assay

Cell viability at starting and ending day of compound treatment was determined by measuring cellular ATP content using the CellTiter-Glo luminescence assay (Promega). CellTiter-Glo reagent was added to each well and luminescence recorded on an Envision plate reader (Perkin Elmer). Luminescence values were used to calculate the inhibition.

### Amplification of PHGDH

Performing TaqMan qPCR for *PHGDH* (3 replicates) using serial dilutions of a known amount of human genomic DNA (Promega, # G3041), then plot the Ct for each dilution vs the log of the concentration to determine the slope of the line which is the efficiency of the PCR.

### Tumor xenografts

Mice were maintained and handled in accordance with Novartis Biomedical Research Animal Care and Use Committee protocols and regulations. The cell number used for *in vivo* xenograft experiments was pre-determined by evaluating the tumor growth latency and doubling time after implanting different number of cells into immuno-compromised mice. The cell number that gives the optimal growth kinetics will be used for the subsequent experiments. MDA-MB-468, HCC1806 and BT20 cells engineered with Tet-inducible shRNA against *PHGDH* were cultured in DMEM and EMEM supplemented with 10% Tet-approved FBS. Mice (6–8 weeks old, n=6-8/each group) were orthotopically inoculated 200 × 10^6^ MDA-MB-468 cells or 8× 10^6^ HCC1806 cells or 3× 10^6^ BT20 cells into the mammary fat pad region. Tumor volume was measured by calipering in two dimensions and calculated as (length × width) / 2. Drug treatment started after implanting when average tumor volume was around 200 mm^3^. Animals received vehicle (5% dextrose, 10 ml/kg, orally, qw) or Doxycycline (25mg/kg, orally, qd) for the duration of the study. At termination of the study, tumor tissues were excised and snap frozen in liquid nitrogen for immunoblotting analyses of biomarkers. Data were expressed as mean ± SEM, and differences were considered statistically significant at *P* < 0.05 by Student *t* test.

### Cell extraction and chemical derivatization of amino acids

Cell pellets were collected and stored at −80 °C until extracted. Extractions were performed in 80% methanol in water kept at −80 °C, and samples were then sonicated in ice-water bath for 10 minutes. Samples were then vortexed until pellets were re-suspended, placed on dry ice for 30 minutes, and then centrifuged at 4 °C at 14,000 RPM for 10 minutes. The supernatant was used for amino acid derivatization. AccQ Tag derivatization kit was purchased from Waters (Milford, MA, USA). Derivatization reagent solution was prepared following the instruction. 10 uL cell extract supernatant was mixed with 70 uL buffer and then mixed with 20 uL reagent solution before incubated at 50 °C for 15 minutes. Then the solution was diluted 10 times by water before LC-MS/MS analysis.

### LC-MS/MS method

An AB Sciex 4000 triple quadrupole mass spectrometer (Foster City, CA, USA) equipped with a Waters I-class UPLC system (Milford, MA, USA) was used for the analysis. The mass spectrometer was operated in the ESI positive ionization multiple reaction monitoring (MRM transition: 276.2171.1 for serine, 278.1171.1 for ^13^C_2_-serine, 279.1171.1 for ^13^C_3_-serine, 246.1171.1 for glycine and 248.1171.1 for ^13^C_2_-glycine) mode with a spray voltage of 4500 V. Ion source gas 1, ion source gas 2 and curtain gas were set at 60, 60, 40 psi, respectively and the source temperature was maintained at 530 °C. Separations were accomplished on a Waters Acquity UPLC HSS T3 Column (2.1x50 mm, 1.8 μm, Milford, MA, USA). The sample chamber was maintained at 4 ^°^C, while the column was kept at 50 °C. The mobile phase consisted of 0.1% formic acid in water (A) and acetronitrile with 0.1% formic acid (B) and was delivered at 0.6 mL/min. The gradient started at 2% of B for 2 minutes, proceeded linearly to 35% of B at 5 minutes and to 95% of B at 5.1 minutes, maintained at 95% of B for 0.9 minute, then returned to initial condition at 6.1 minutes. The total run time is 7.5 minutes.

### Statistics

Statistical analyses were performed by using Student *t* Test (*: *p*<0.05, **: *p*<0.01) (GraphPad Prism v6.01 software for Windows, GraphPad Software, San Diego, CA USA).

## Supplemental Figures


