# Supplementary material for: Phosphoglycerate dehydrogenase is dispensable for breast tumor maintenance and growth
Source: Oncotarget. 2013 Nov 26;4(12):2502–11. doi: 10.18632/oncotarget.1540 (PMC3926844; doi:10.18632/oncotarget.1540)
Supplement: Supplementary file 1 [file oncotarget-04-2502-s001.pdf]

Phosphoglycerate dehydrogenase is dispensable for breast tumor maintenance and growth - Chen et al

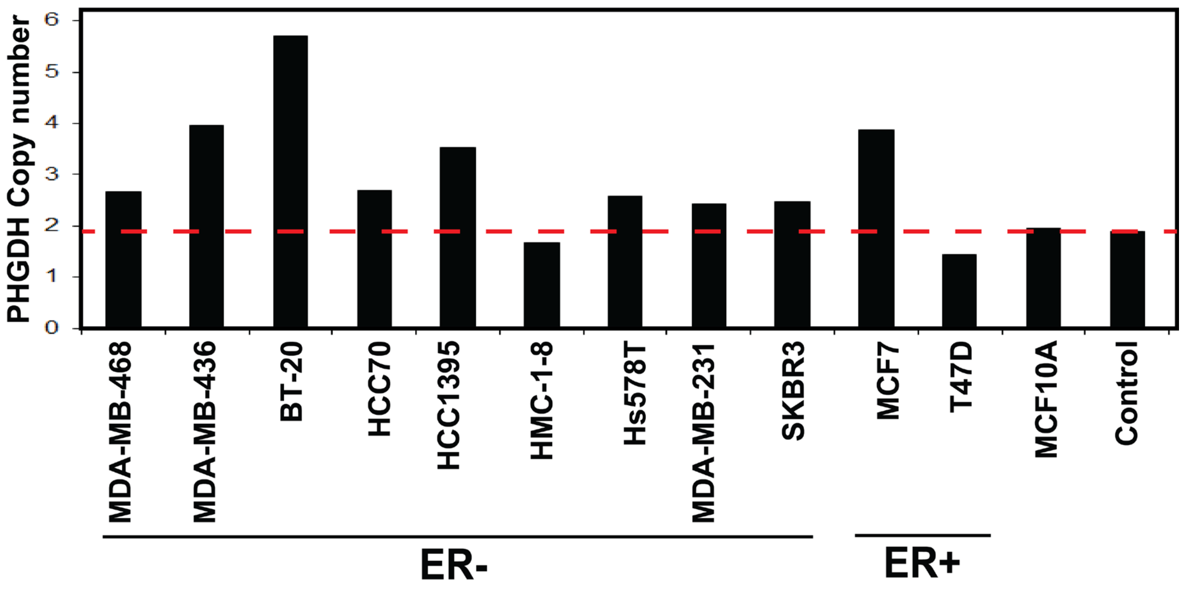

Supplementary Figure S1: PHGDH copy number gain in human breast cancer cells. Genomic DNA copies of PHGDH were measured among 12 human breast cancer cell lines and 1 human normal breast cell line by PCR

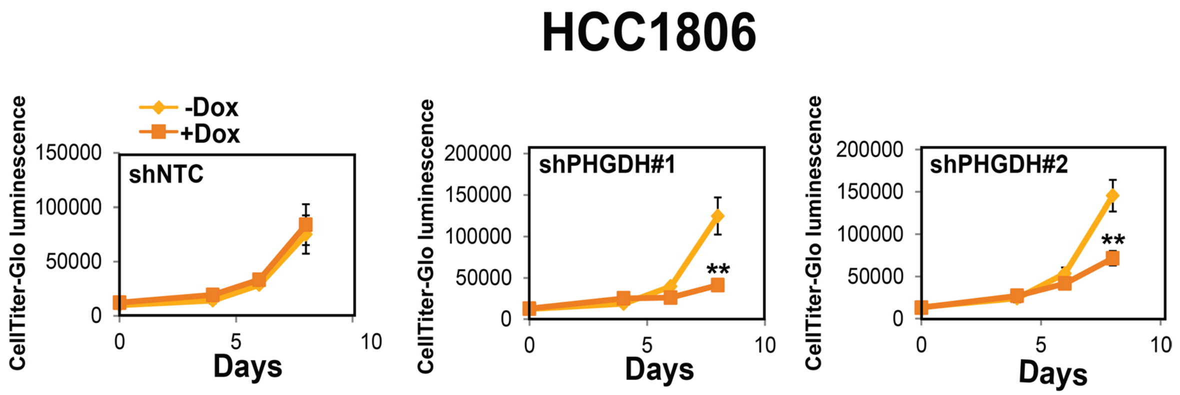

Supplementary Figure S2: The proliferation of HCC1806 with or without PHGDH knockdown. shNTC or shPHGDH transduced cancer cells were treated with or without Doxycycline for different days. Relative cell growth (average of at least 3 independent experiments) was measured by CellTiter-Glo.

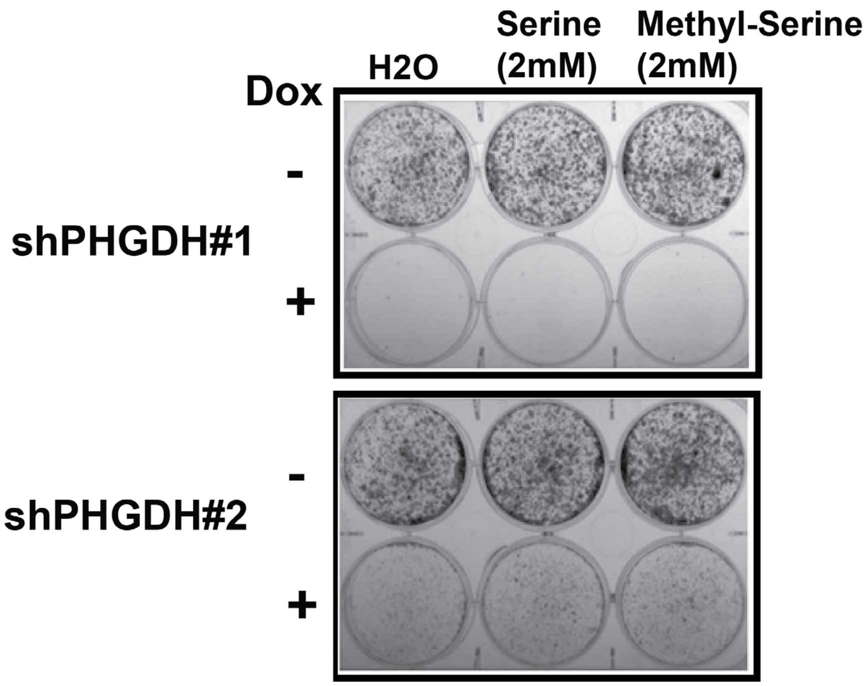

.Supplementary Figure S3: Serine or methyl-serine didn't rescue the cell growth medicated by PHGDH knockdown. Cell colony formation assay of PHGDH knockdown in BT-20 cells treated with serine or methyl-serine. shNTC or shPHGDH transduced cells were treated with or without Doxycycline under the treatment of serine or methyl-serine for 14 days.
